# Draft genome sequence of a multidrug-resistant *Mycobacterium tuberculosis* clinical isolate, 3184-KZ

**DOI:** 10.1128/MRA.00863-23

**Published:** 2023-11-15

**Authors:** Dana Auganova, Sabina Atavliyeva, Akmaral Akisheva, Anna Tsepke, Pavel Tarlykov

**Affiliations:** 1 Core Facility of Genomics and Proteomics, National Center for Biotechnology, Astana, Kazakhstan; 2 Clinical Bacteriological Laboratory, City Center for Phthisiopulmonology of the Akimat of Astana, Astana, Kazakhstan; University of Maryland School of Medicine, Baltimore, Maryland, USA

**Keywords:** *Mycobacterium tuberculosis*, drug resistance, whole-genome sequencing, Kazakhstan

## Abstract

*Mycobacterium tuberculosis* is an obligate aerobic bacterium that is the causative agent of tuberculosis. Here, we announce the draft genome sequence of multidrug-resistant *Mycobacterium tuberculosis* clinical isolate, 3184-KZ, from a patient with infiltrative pulmonary tuberculosis from Kazakhstan.

## ANNOUNCEMENT

The most clinically relevant species of the *Mycobacterium tuberculosis* complex is *Mycobacterium tuberculosis*. Multidrug-resistant tuberculosis (MDR-TB) is caused by *M. tuberculosis* strains, resistant to at least rifampicin and isoniazid (1). Half a million patients are diagnosed with MDR-TB each year worldwide, as stated by the latest TB report by the WHO (2). According to the report, Kazakhstan is among the 30 countries with the highest rates of MDR-TB. Whole-genome sequencing (WGS) and other molecular techniques for TB control were recently implemented in Kazakhstan (3–9).

*Mycobacterium tuberculosis* clinical isolate 3184-KZ was isolated in 2014 from the sputum of a patient with infiltrative pulmonary tuberculosis in Astana, Kazakhstan. The study was approved by the Ethics Committee of the National Center for Biotechnology (Protocol No. 07, approved 4 February 2013). A Bactec MGIT 960 liquid medium-based culture system (Becton, Dickinson) was used for the cultivation of *M. tuberculosis*. The isolate was phenotypically resistant to isoniazid, rifampin, streptomycin, and ethambutol, as confirmed by drug susceptibility testing (DST) using a Bactec MGIT 960 system as per the manufacturer’s protocol. DNA was extracted using the cetyltrimethylammonium bromide (CTAB) procedure (10). The quality of DNA was checked using a Qubit dsDNA High Sensitivity Assay Kit (Thermo) and a Qubit 2.0 Fluorometer (Thermo). Ten nanograms of the total *M. tuberculosis* 3184-KZ genomic DNA was used to prepare a barcoded library with a Nextera DNA Flex Library Prep kit (Illumina). A MiSeq platform (Illumina) and Reagent Kit v3 (Illumina) were used to carry out paired-end sequencing. The genome of *M. tuberculosis* isolate 3184-KZ has a length of 4,123,173 bp. It was sequenced with 81-fold coverage, a mean read length of 154 bp, and a G+C content of 65.60%. A total of 3,183,265 reads were produced. The quality check of the reads was performed using FastQC v.0.11.9 (11). A total of 66 contigs, with an *N*_50_ value of 94,099 bp, were *de novo* assembled using SPAdes v.3.13.1 (12). The final assemblies were annotated with the NCBI Prokaryotic Genome Annotation Pipeline (PGAP) v.6.6 (13). Default parameters were used for all software. The annotation of the 3184-KZ genome with PGAP identified a total of 3,940 genes, of which 3,889 were predicted coding sequences. Additionally, there were 3 rRNAs, 45 tRNAs, and 3 noncoding RNA (ncRNA) genes.

The BWA-MEM algorithm and SAMtools v.1.0.18 were used for a generalized mapping procedure (14, 15). The sorted alignment file was saved in the binary alignment format (BAM). Single-nucleotide polymorphisms (SNPs) were called by GATK UnifiedGenotyper pipeline v.4.1.4 using the *M. tuberculosis* H37Rv genome (NC_000962.3) (16). A phylogenetically informative SNP in position 797736 (C/T) specific to lineage 2.2.1 (L2/Beijing) was identified. The 3184-KZ genome harbors high-confidence mutations in the *katG* gene (Ser315Thr), *rpoB* (Ser450Leu), *rpsL* (Lys43Arg), *embB* (Met306Val), and *pncA* (82_517del) associated with drug resistance to isoniazid, rifampin, streptomycin, ethambutol, and pyrazinamide, respectively (17).

A comparison of the 3184-KZ genome with the available genomic data from other local drug-resistant isolates will help reveal genetic differences responsible for its pathogenicity and transmission patterns.

## Data Availability

This Whole Genome Shotgun project has been deposited at DDBJ/ENA/GenBank under the accession number JAVLTY000000000. The version described here is the first version. The raw data from BioProject number PRJNA1013742 were submitted to the NCBI Sequence Read Archive under experiment accession number SRR25936541.

## References

[1] World Health O. 2020. Meeting report of the WHO expert consultation on the definition of extensively drug-resistant tuberculosis. World Health Organization, Geneva.

[2] World Health O. 2022. Global tuberculosis report 2022. World Health Organization, Geneva.

[3] Daniyarov A, Akhmetova A, Rakhimova S, Abilova Z, Yerezhepov D, Chingissova L, Bismilda V, Takenov N, Akilzhanova A, Kairov U, Kozhamkulov U. 2023. Whole-genome sequence-based characterization of pre-XDR M. tuberculosis clinical isolates collected in Kazakhstan. Diagnostics (Basel) 13:2005. doi:10.3390/diagnostics13122005 37370900 PMC10296843

[4] Daniyarov A, Molkenov A, Rakhimova S, Akhmetova A, Yerezhepov D, Chingissova L, Bismilda V, Toksanbayeva B, Rakisheva A, Akilzhanova A, Kozhamkulov U, Kairov U. 2021. Genomic analysis of multidrug-resistant Mycobacterium tuberculosis strains from patients in Kazakhstan. Front Genet 12:683515. doi:10.3389/fgene.2021.683515 34858467 PMC8630622

[5] Tarlykov P, Atavliyeva S, Alenova A, Ramankulov Y. 2020. Draft genome sequence of an extensively drug-resistant Mycobacterium tuberculosis clinical isolate, 3485_MTB, from Nur-Sultan, Kazakhstan. Microbiol Resour Announc 9:e00025-20. doi:10.1128/MRA.00025-20 32139580 PMC7171203

[6] Tarlykov P, Atavliyeva S, Alenova A, Ramankulov Y. 2020. Genomic analysis of Latin American-Mediterranean family of Mycobacterium tuberculosis clinical strains from Kazakhstan. Mem Inst Oswaldo Cruz 115:e200215. doi:10.1590/0074-02760200215 32965331 PMC7508292

[7] Klotoe BJ, Kacimi S, Costa-Conceicão E, Gomes HM, Barcellos RB, Panaiotov S, Haj Slimene D, Sikhayeva N, Sengstake S, Schuitema AR, Akhalaia M, Alenova A, Zholdybayeva E, Tarlykov P, Anthony R, Refrégier G, Sola C. 2019. Genomic characterization of MDR/XDR-TB in Kazakhstan by a combination of high-throughput methods predominantly shows the ongoing transmission of L2/Beijing 94-32 central Asian/Russian clusters. BMC Infect Dis 19:553. doi:10.1186/s12879-019-4201-2 31234780 PMC6592005

[8] Abeldenov S, Talhaoui I, Zharkov DO, Ishchenko AA, Ramanculov E, Saparbaev M, Khassenov B. 2015. Characterization of DNA substrate specificities of apurinic/apyrimidinic endonucleases from Mycobacterium tuberculosis. DNA Repair (Amst) 33:1–16. doi:10.1016/j.dnarep.2015.05.007 26043425

[9] Kozhamkulov U, Akhmetova A, Rakhimova S, Belova E, Alenova A, Bismilda V, Chingissova L, Ismailov S, Ramanculov E, Momynaliev K. 2011. Molecular characterization of Rifampicin- and isoniazid-resistant Mycobacterium tuberculosis strains isolated in Kazakhstan. Jpn J Infect Dis 64:253–255. doi:10.7883/yoken.64.253 21617314

[10] Wilson K. 2001. Preparation of genomic DNA from bacteria. Curr Protoc Mol Biol Chapter 2:Unit. doi:10.1002/0471142727.mb0204s56 18265184

[11] Andrews S. 2019. FastQC: a quality control tool for high throughput sequence data. Available from: http://www.bioinformatics.babraham.ac.uk/projects/fastqc

[12] Nurk S, Bankevich A, Antipov D, Gurevich AA, Korobeynikov A, Lapidus A, Prjibelski AD, Pyshkin A, Sirotkin A, Sirotkin Y, Stepanauskas R, Clingenpeel SR, Woyke T, McLean JS, Lasken R, Tesler G, Alekseyev MA, Pevzner PA. 2013. Assembling single-cell genomes and mini-metagenomes from chimeric MDA products. J Comput Biol 20:714–737. doi:10.1089/cmb.2013.0084 24093227 PMC3791033

[13] Tatusova T, DiCuccio M, Badretdin A, Chetvernin V, Nawrocki EP, Zaslavsky L, Lomsadze A, Pruitt KD, Borodovsky M, Ostell J. 2016. NCBI prokaryotic genome annotation pipeline. Nucleic Acids Res 44:6614–6624. doi:10.1093/nar/gkw569 27342282 PMC5001611

[14] Li H, Durbin R. 2010. Fast and accurate long-read alignment with burrows-wheeler transform. Bioinformatics 26:589–595. doi:10.1093/bioinformatics/btp698 20080505 PMC2828108

[15] Li H, Handsaker B, Wysoker A, Fennell T, Ruan J, Homer N, Marth G, Abecasis G, Durbin R. 2009. The sequence alignment/map format and Samtools. Bioinformatics 25:2078–2079. doi:10.1093/bioinformatics/btp352 19505943 PMC2723002

[16] McKenna A, Hanna M, Banks E, Sivachenko A, Cibulskis K, Kernytsky A, Garimella K, Altshuler D, Gabriel S, Daly M, DePristo MA. 2010. The genome analysis toolkit: a mapreduce framework for analyzing next-generation DNA sequencing data. Genome Res. 20:1297–1303. doi:10.1101/gr.107524.110 20644199 PMC2928508

[17] World Health O. 2021. Catalogue of mutations in Mycobacterium tuberculosis complex and their association with drug resistance: supplementary document. World Health Organization, Geneva.

